# Morphology does not covary with predicted behavioral correlations of the domestication syndrome in dogs

**DOI:** 10.1002/evl3.168

**Published:** 2020-04-10

**Authors:** Christina Hansen Wheat, Wouter van der Bijl, Christopher W. Wheat

**Affiliations:** ^1^ Department of Zoology Stockholm University Stockholm SE‐10961 Sweden; ^2^ Department of Zoology and Biodiversity Research Centre University of British Columbia Vancouver BC V6T 1Z4 Canada

**Keywords:** Behavior, domestication, morphological evolution

## Abstract

Domesticated animals display suites of altered morphological, behavioral, and physiological traits compared to their wild ancestors, a phenomenon known as the domestication syndrome (DS). Because these alterations are observed to co‐occur across a wide range of present day domesticates, the traits within the DS are assumed to covary within species and a single developmental mechanism has been hypothesized to cause the observed co‐occurrence. However, due to the lack of formal testing it is currently not well‐resolved if the traits within DS actually covary. Here, we test the hypothesis that the presence of the classic morphological domestication traits white pigmentation, floppy ears, and curly tails predict the strength of behavioral correlations in support of the DS in 78 dog breeds. Contrary to the expectations of covariation among DS traits, we found that morphological traits did not covary among themselves, nor did they predict the strength of behavioral correlations among dog breeds. Further, the number of morphological traits in a breed did not predict the strength of behavioral correlations. Our results thus contrast with the hypothesis that the DS arises due to a shared underlying mechanism, but more importantly, questions if the morphological traits embedded in the DS are actual domestication traits or postdomestication improvement traits. For dogs, it seems highly likely that strong selection for breed specific morphological traits only happened recently and in relation to breed formation. Present day dogs therefore have limited bearing of the initial selection pressures applied during domestication and we should reevaluate our expectations of the DS accordingly.

**Impact Summary**

Domesticated animals display suites of altered morphological, behavioral, and physiological traits compared to their wild ancestors, a phenomenon known as the domestication syndrome (DS). Classic morphological “domestication traits” are white pigmentation, floppy ears, and curly tails and reduced aggression and increased sociability are among the expected behavioral changes caused by domestication. Because these alterations are observed to co‐occur across a wide range of present day domesticates, the traits within the DS are assumed to covary within species and a single developmental mechanism has been suggested to cause the DS. However, very few studies have tested whether the traits within DS actually covary. The domestic dog has been argued to be the only species expressing the full DS, but dogs have been bred for highly breed‐specific morphological and behavioral traits and key behavioral and morphological DS traits do not appear to occur simultaneously across breeds. It is therefore unclear if we should expect the DS in dogs. Here, we investigated the relationship between classic morphological DS traits and behavioral correlations in the DS in 78 dog breeds. Contrary to the expectations, we found that morphological traits did not covary among themselves, nor did they predict the strength of behavioral correlations among dog breeds. Further, the number of morphological traits in a breed did not predict the strength of behavioral correlations. Our results thus contrast with the hypothesis that the DS arises due to a shared underlying mechanism, but more importantly, questions if the morphological traits embedded in the DS are actual domestication traits or postdomestication improvement traits. For dogs, it seems highly likely that strong selection for breed specific morphological traits only happened recently and in relation to breed formation. Present day dogs therefore have limited bearing of the initial selection pressures applied during domestication and we should reevaluate our expectations of the DS accordingly.

Domesticated animals display suites of altered morphological, behavioral, and physiological traits compared to their wild ancestors, a phenomenon known as the domestication syndrome (DS). Key examples of components in the DS are increased tameness, reduced brain size, white pigmentation, and decreased hypothalamic‐pituitary‐adrenal axis reactivity (Kruska 1996; Driscoll et al. 2009; Trut et al. 2009). Because these alterations are observed to co‐occur across a wide range of present day domesticates, such as dogs (*Canis familiaris*), cats (*Felis catus*), rabbits (*Oryctolagus cuniculus*), horses (*Equus caballus*), and pigs (*Sus scrofa*) (Sánchez‐Villagra et al. 2016), the traits within the DS are assumed to covary within species (Trut 1998; Trut et al. 2009). Domestication experiments have demonstrated that selection for tame behavior alone can produce the myriad changes seen in the DS (Belyaev et al. 1985; Trut et al. 2009), and recent evidence suggests that long‐term indirect selection for tameness can produce DS traits in free‐living populations as well (Geiger et al. 2018). Although the mechanistic origin of the DS is currently unresolved, these findings have nurtured the hypothesis that the convergent patterns seen across domesticated species arise via a singular developmental mechanism such as altered neuroendocrine control of ontogenesis (Belyaev 1979), or neural crest deficit during embryogenesis (Wilkins et al. 2014). Both of these influential studies have led to the general assumption that morphological changes, such as white pigmentation, floppy ears, and curly tails, have arisen as by‐products of the physiological alterations caused by selection upon behavior (Wilkins et al. 2014).

These two independent hypotheses suggesting that the DS is founded in single developmental mechanism offer a coherent, logical, parsimonious, and satisfying explanation for the observed covariation among DS traits. However, traits of the DS are not fully consistent with such hypotheses and recently support for the existence of a DS in animals has been called into question (Lord et al. 2019). First, DS traits are not evenly distributed among domesticated animals (Sánchez‐Villagra et al. 2016). Second, even though rat (*Rattus norvegicus*) lines selected for tameness have an increased frequency of white spots (Trut et al. 2000), a quantitative trait locus (QTL) study of >700 rats found neither any overlap between QTLs for tameness and pigmentation nor any correlation between these two phenotypes among the F2 offspring (Albert et al. 2009). This is unexpected if these DS traits originate from a shared mechanism, such as an altered ontogenesis (Belyaev 1979) or neural crest deficit (Wilkins et al. 2014). Specifically, syndrome traits with a shared underlying mechanism should be difficult to decouple compared to statistically correlated traits with independent origins (sensu Sih et al. 2004). Finally, recent genomic studies in horses (Librado et al. 2017), foxes (*Vulpes vulpes*; Wang et al. 2018), dogs (Pendleton et al. 2018), and cats (Montague et al. 2014) find signatures of domestication selection pressures in genes associated with neural crest development. Although these findings are argued to support the neural crest hypothesis (i.e., additive effects of genes causing neural crest cell hypofunction, which in turn is the singular developmental basis of pleiotropic effects manifesting as the DS), these genes are only a subset of many showing selective signatures during domestication. Thus, although it is generally assumed that DS traits covary, possibly due to a single developmental mechanism, further quantitative testing of this hypothesis is warranted.

Recently, a formal test of covariance among behavioral DS traits was conducted among dog breeds. In their study of the behavioral component of the DS in more than 76,000 dogs, Hansen Wheat et al. (2019) demonstrated that although correlations among fear, aggression, sociability, and playfulness were stronger in ancient breeds, these correlations were weaker or had been decoupled in modern breeds. However, this study focused only upon behavior, which was likely the focal trait in dog domestication (sensu Belyaev et al. 1985; Trut et al. 2009). Studies investigating the covariation of morphological traits, either among themselves or with the expected behavioral correlations of the DS, remain absent from the literature. Therefore, formal investigations of the predicted expectations of how behavioral and morphological components of domestication arise is needed if we are to further our understanding of the DS.

Among domesticates, the dog has been argued to be the only species expressing the full DS (Sánchez‐Villagra et al. 2016). Dogs have been bred for highly breed‐specific morphological and behavioral traits (Svartberg 2006; Mehrkam and Wynne 2014), which is illustrated by the extreme phenotypic variation expressed among the more than 400 present day dog breeds (Lindblad‐Toh et al. 2005; Parker et al. 2017). Although key DS traits of behavior and morphology do not qualitatively appear to occur simultaneously across breeds (Sánchez‐Villagra et al. 2016), this has never been tested quantitatively. Furthermore, although dogs express a range of traits not present in wolves (Parker et al. 2009; Larson et al. 2014), it is currently not well resolved if present day dog traits are original domestication traits, that is, traits evolved as a consequence of altered selection pressures during the initial stages of domestication, or so‐called improvement traits that have been secondarily enhanced postdomestication during breed formation (sensu Olsen and Wendel 2013, Larson and Fuller 2014; Lord et al. 2019).

With modern breeds created from intense breeding efforts only within the last 150‐200 years (Lindblad‐Toh et al. 2005; vonHoldt et al. 2010), it is possible that modern dogs provide a suboptimal basis for the expectations embedded in the DS. Indeed, as noted earlier, modern dogs lack the strong behavioral correlations expected of the DS (Hansen Wheat et al. 2019). Nonetheless, because the foundation for the DS hypothesis is based on extant domesticates, it remains unclear if we should expect the expression of the DS to vary across different stages of domestication. Archaeological findings of early dogs provide limited information on morphology (i.e., skeletal features), and none on behavior, which impairs our ability to compare trait expression in dogs at different stages of domestication. Prebreed formation domesticated dogs, that is, village dogs, could be very informative, but unfortunately, the only nonadmixed village dog populations identified to date are found in Borneo (Shannon et al. 2015) and have not been studied behaviorally. However, a small group of present day dogs can be categorized as ancient breeds due to their (a) detectable admixture with wolf, which is not present in modern breeds, and (b) an origin about 500 years ago (Lindblad‐Toh et al. 2005; vonHoldt et al. 2010). Certainly, ancient breeds are expected to have improvement traits, but importantly, these breeds have been shown to have stronger behavioral correlations expected of the DS compared to modern breeds (Hansen Wheat et al. 2019). While acknowledging that (a) dog phylogenies inherently are associated with uncertainties due to the domestication history of the dog (Tonoike et al. 2015) and (b) ancient breeds are an imperfect proxy for early domestic dogs, ancient breeds are arguably the only available representatives for earlier stages of dog domestication. Thus the division of ancient and modern breeds provides an opportunity for temporal comparisons among dogs on a domestication time scale.

Here, we test the hypothesis that the presence of morphological traits associated with the DS predict the strength of behavioral correlations in support of the DS in dogs. For the morphological component of our study, we focused upon variation in the traits white pigmentation, floppy ears, and curly tails, which have been referred to as morphological markers of domestication (Trut et al. 2009). For the behavioral component, we used estimates of effect sizes for behavioral correlations associated with the DS, derived from data extracted from the Swedish Kennel Club's database on 76,158 dogs completing a highly standardized behavioral test battery (Hansen Wheat et al. 2019). We then matched these effect sizes of behavioral correlations with our estimates of morphological traits from the 78 breeds completing the behavioral test. We further added a temporal component by assessing seven ancient and 71 modern breeds separately, referring to previously used divisions of these breed categories (Tonoike et al. 2015; Smith et al. 2017; Hansen Wheat et al. 2019) based on recent dog phylogenies (Lindblad‐Toh et al. 2005; vonHoldt et al. 2010; Parker et al. 2017). As predicted by the DS, we expected that the presence of white pigmentation, floppy ears, and curly tails would co‐vary among breeds. Additionally, we expected that the presence or absence of these morphological traits would predict the strength of behavioral correlations of the DS. That is, we expected stronger behavioral correlations of the DS when morphological traits of the DS are present. We further predicted that behavioral correlations would be stronger with the number of morphological traits present.

## Methods

### BREED CATEGORIES

We based our study on the 78 dog breeds used in a recent study to test behavioral correlations within the DS (Hansen Wheat et al. 2019). Of the 78 breeds, seven were ancient breeds and 71 modern breeds (Lindblad‐Toh et al. 2005; vonHoldt et al. 2010; Tonoike et al. 2015; Smith et al. 2017). This difference in sample sizes between breed groups does not reflect a lack of sampling effort, but the natural limitation of only few breeds being categorized as ancient. In recent dog phylogenies (Parker et al. 2004; vonHoldt et al. 2010; Larson et al. 2012; Parker et al. 2017), some discrepancy exists for the placement of the breeds Samoyed and Saluki. Samoyed has been classified as ancient by vonHoldt et al. (2010) with good bootstrap support and the breed clusters with ancient breeds in Parker et al. (2017). We therefore classify this breed as ancient. Although Parker et al. (2017) placed the Saluki as a modern breed, Parker et al. (2004), vonHoldt et al. (2010), and Larson et al. (2012) all categorize the Saluki as ancient. Based on the good bootstrap support for the placement of the Saluki in vonHoldt et al. (2010), we chose to include the Saluki as an ancient breed. However, recognizing that the categorization of the Saluki remains associated with uncertainty, we repeated all analyses with the Saluki as a modern breed. This did not affect our conclusions (Table S1). Last, our study was based on a previous study using the same categorization of ancient and modern breeds to investigate the behavioral components of the DS (Hansen Wheat et al. 2019), thereby warranting the use of the same breed categorization in our analyses here between morphology and these same behavioral traits.

### MORPHOLOGICAL ASSESSMENT

**Figure 1:**
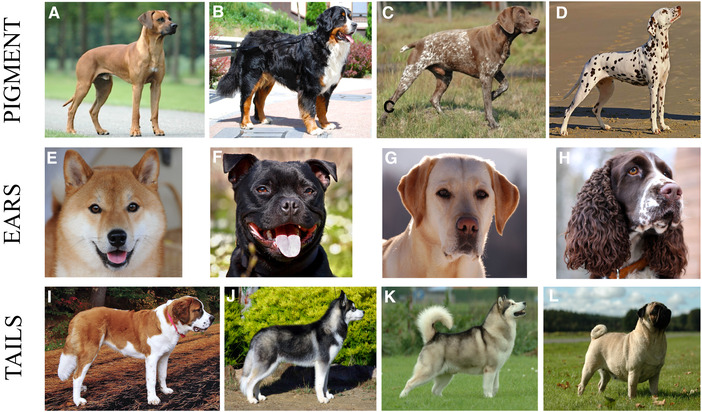
Morphological assessments. Examples of morphological variation across dog breeds and how this was taken into account when assessing the presence and absence of morphological traits in the DS. White pigmentation (pigment; A‐D): Breeds where a small white spot or a few white hairs on the chest is tolerated or undesirable, here illustrated in a Rhodesian Ridgeback (A), were categorized as not having white pigmentation in the conservative assessment, but as having white pigmentation in the relaxed assessment. The presence of white pigmentation varies across breeds in size, shape, and placement as illustrated in Bernese Mountain Dog (B), German Short‐haired Pointer (C), and Dalmatian (D). Floppy ears (ears; E‐H): Floppiness of ears is binary and erect ears, as illustrated in the Shiba (E), can never be floppy. Other examples of breeds with erect ears are Siberian Husky (J) and Alaskan Malamute (K). The floppiness of ears can be graduated as illustrated by the Staffordshire Bull Terrier (F), Labrador Retriever (G), and English Springer Spaniel (H). Any degree of floppiness of the ears was assessed as presence of floppy ears. Curly tail (tails; I‐L): Breeds, such as the St. Bernard (I), with tails hanging straight down and never carry their tail in a curl, curve, hook, sickle, sabre of J shaped, express the absence of a curly tail (both assessments). Many breeds carry their tail in a curl, curve, hook, sickle, sabre of J shaped fashion but can also let their tail straight down, here illustrated by Siberian Husky with a letdown tail (J) and an Alaskan Malamute with a tail carried in a curl (K). For the conservative assessment, such breeds were categorized as not having curly tails, whereas they were categorized as having curly tails in the relaxed assessment. Other examples of breeds categorized like this are Rhodesian Ridgeback (A) and Dalmatian (D). A few breeds, such as Pugs (L), express the presence of a permanent curly tail (both assessments). All photos are from wikicommons; please see references for specific credits.

**Figure 2:**
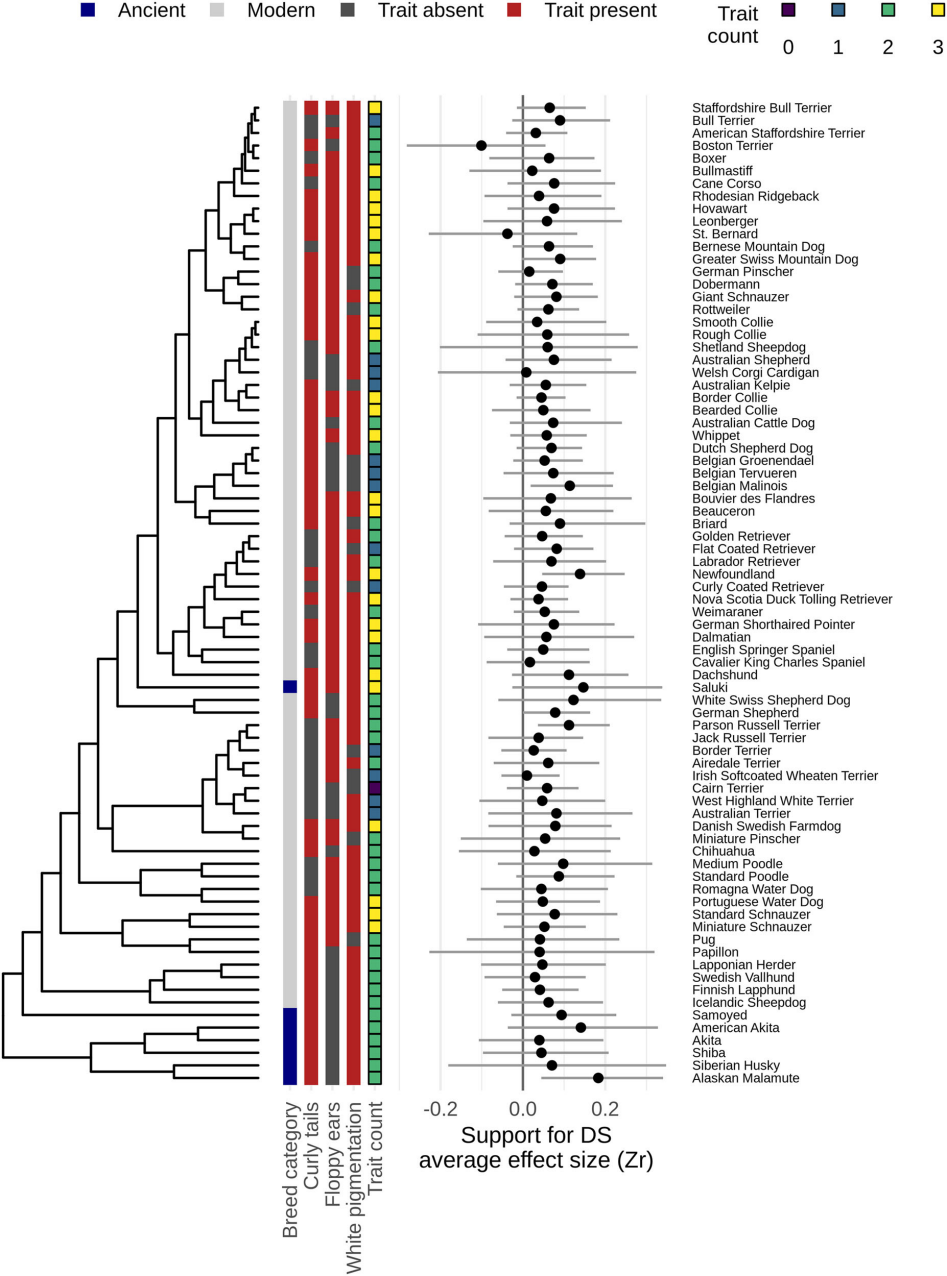
Morphological scores placed onto the latest dog phylogeny. Morphological scores based on the presence or absence of curly tail, floppy ears, and white pigmentation (relaxed assessment), and average effect sizes for behavioral correlations in ancient and modern dog breeds placed onto the latest dog phylogeny (Parker et al. 2017). Average effect sizes were calculated by separate meta‐analytic models per breed (not used for inference), and posterior means ±95% credible intervals are depicted.

We carefully inspected the breed standards for those 78 breeds by consulting the Fédèration Cynologique Internationale (http://www.fci.be), the world's largest federation of kennel clubs, to assess the presence or absence of our three chosen morphological traits: white pigmentation, floppy ears, and curly tails. It was not possible to consider within‐breed variation deviating from breed standards, but as an attempt to assess such an effect, we used both relaxed and conservative assessments of the three morphological traits (Figs. 1, 2, and S1). We defined white pigmentation as any form of white pigmentation in the breed, regardless of its placement or shape. We also classified dogs with a white base color, such as Dalmatians and Samoyeds, to express white pigmentation. Breeds where “white” was not mentioned in the coat color description, such as Dobermann and Rottweiler, were assessed as not having white pigmentation. For our conservative assessment of white pigmentation, only breeds specifically described to have a white base color or characteristic white coloration, or breeds where some versions have white pigmentation (such as Schnauzers) were included. Breeds where a small white spot or a few white hairs are “tolerated” or “undesirable” were not included as having white pigmentation in our conservative assessment. For the relaxed assessment, we included breeds in which small white spots or a few white hairs, for instance on the chest, are “tolerated” or “undesirable” (Fig. 1A‐D). Floppy ears were assessed based on whether a breed has ears that are either erect or to some degree floppy (i.e., from just the tip to hanging straight down; Fig. 1E‐H). Thereby the presence or absence of floppy ears was assessed as a completely binary trait, and did not differ between the relaxed and conservative assessments. For our conservative assessment of curly tails, only breeds described to specifically have their tail in a permanent curl, that is, the tail never hangs down, as seen in Pugs, were included. For the relaxed assessment breeds that are described to carry their tail in a “curl,” “hook,” “sabre,” “sickle,” or “J”, and even breeds carrying their tails in the slightest “curve,” but can let their tails straight down were assessed as having curly tails (Fig. 1I‐L). Breeds where the words “curl,” “hook,” “sabre,” “sickle,” “J,” and “curve” were not included in the description of the tail were assessed as not having a curly tail in either assessment.

### BEHAVIORAL ASSESSMENT

For the behavioral component of our study, we used the dataset presented in Hansen Wheat et al. (2019), in which the strength and direction of behavioral correlations among aggression, fearfulness, sociability, and playfulness across the 78 dog breeds were investigated. Behavioral data were provided by the Swedish Kennel Club for dog completing the Dog Mentality Assessment, a highly standardized behavioral test for dogs in Sweden, in which only purebred dogs with a full pedigree are allowed to participate. We refer to Hansen Wheat et al. (2019) for a full description of the methods used to estimate the effect sizes for these behavioral correlations.

### STATISTICAL ANALYSES

To evaluate the relationship between breed morphology and agreement with the DS hypothesis, we assessed the correlation between our morphology scores, treated as dichotomous variables. First, we estimated the phi coefficient (*ϕ*) for presence/absence of each trait in pairwise combinations with significance determined using Fisher's Exact Test, as implemented in the xtab_statistics function of the sjstats package version 0.17.5 (Lüdeke 2019). Second, we repeated this analysis using a Pearson's product‐moment correlation with similar results. Third, we assessed whether the presence/absence of traits was correlated while taking into account phylogenetic correction, using a pairwise bionomial phylogenetic glm.

To evaluate the relationship between breed morphology and agreement with the DS hypothesis, as quantified by the strength and direction of behavioral correlations, we used a meta‐analytic model. It is a multi‐level model that uses the 1326 observed correlation coefficients (Hansen Wheat et al. 2019), and their associated uncertainty, as the dependent variable. These correlations test multiple behavioral predictions by the DS, such as a positive association between sociability and playfulness, or a negative association between sociability and aggression (Trut et al. 2009; Himmler et al. 2013). The correlations test six such DS predictions. For some predictions, multiple correlations per breed were measured, because the Dog Mental Assessment test provided multiple measurements for aggression and fearfulness. A total of 17 correlations were obtained per breed. Therefore, we treat the DS as a nested compound hypothesis, with six predicted associations and 17 correlations. We aligned the sign of the correlations with the predicted directions, that is, we flipped the sign of correlations expected to be negative, so that positive effect sizes represent support in favor of the DS.

To account for this nested structure, we included group‐level effects that allow the support for the DS to vary between the different predicted associations and the measured correlations. We additionally included group‐level effects of morphology for the associations and correlations, so that the moderating effect of morphological traits could be stronger or weaker depending on what behavioral correlations were measured. Because each breed was represented by multiple correlations, we included a group‐level intercept for breed. And because breeds are nonindependent due to shared ancestry (Felsenstein 1985), an additional group‐level effect was added with the expected covariance matrix of the phylogeny. Morphology was modeled as three additive binary effects, one each for the presence or absence of white pigmentation, floppy ears, and curly tails. We implemented the models in the probabilistic programming language Stan (Carpenter et al. 2017), using the interfacing R (R Core Team 2019) package brms (Bürkner 2017, 2018). In brms syntax, the models were of the form: *Zr | se(vi, sigma = TRUE) ∼ breed_category + pigmentation + ears + tails + (1 + breed_category + pigmentation + ears + tails || prediction/correlation) + (1 | breed) + (1 | phylogeny)*, where *Zr* is the *z*‐transformed correlation coefficients, vi is the measurement error, and sigma = TRUE allows for the estimation of the residual standard deviation.

To explore whether the relationship between the morphological characters and behavioral correlations was different for modern and ancient breeds, we evaluated a model that also included the interactions between the morphology and breed category terms. This model was evaluated by comparing it to the simpler model described above using leave‐one‐out (LOO) cross‐validation (Vehtari et al. 2017). As the interaction model did not provide a clear improvement in predictive accuracy (difference in log pointwise predictive probability: 0.088, standard error: 0.186), inference is based on the simpler model.

Inference about the effects of morphology was based on two approaches. We used the posterior distributions for the parameters directly to evaluate the role of the three morphological traits separately. Second, we assessed the role of the number of morphological traits (regardless of which trait) by calculating the estimated mean response for each trait combination, and then calculating the marginal mean for a breed having 0, 1, 2, or 3 traits present.

Posterior distributions for the parameters were obtained through Markov chain Monte Carlo (MCMC) sampling, using 16 chains of 2000 iterations each, of which 1000 were warmup. We adjusted the target average proposal acceptance probability to 0.995 and the maximum tree depth to 20 to eliminate any divergent transitions. For population level effects, we used the default weakly informative student‐*t* prior with a mean of 0, scale parameter of 10, and three degrees of freedom. The same prior was used for standard deviations of group‐level effects and the residual standard deviation, but there it was restricted to be nonnegative. Trace plots indicated that the chains were well mixed, and we obtained an effective sample size of more than 2500 for all parameters. The largest $\hat{R}$ was 1.01, indicating convergence.

All analyses were done for both relaxed and conservative assessments of morphological traits. Results for the two different assessments were qualitatively similar, and below we present the results for the relaxed assessment (see Table S2 and Fig. S1 for results for the conservative assessment)

## Results

We placed the morphological traits and average effect sizes for behavioral correlations onto the latest dog phylogeny (Parker et al. 2017), revealing large variation among breeds in both our morphological and behavioral traits (Fig. 2; for conservative assessments see Table S2 and Fig. S1).

First, we used three different methods to test whether the presence of morphological DS traits covaries among themselves. Neither phi coefficients (*φ*), Pearson's product‐moment correlation (*t*), nor phylogenetically corrected correlations (*z*) for the three morphological traits produced significant results: white pigmentation versus floppy ears (*φ* = 0.172, *P*_φ_ = 1; *t* = –0.115, *P_t_* = 0.909; *z* = –0.080, *P_z_* = 0.937), white pigmentation versus curly tail (*φ* = 0.013, *P_φ_* = 1; *t* = –5.7071^−20^, *P_t_* = 1; *z* = 0.653, *P_z_* = 0.514), floppy ears versus curly tail (*φ* = 0, *P_φ_* = 0.2063; *t* = –1.5176, *P_t_* = 0.1333; *z* = –0.49807, *P_z_* = 0.618).

**Figure 3:**
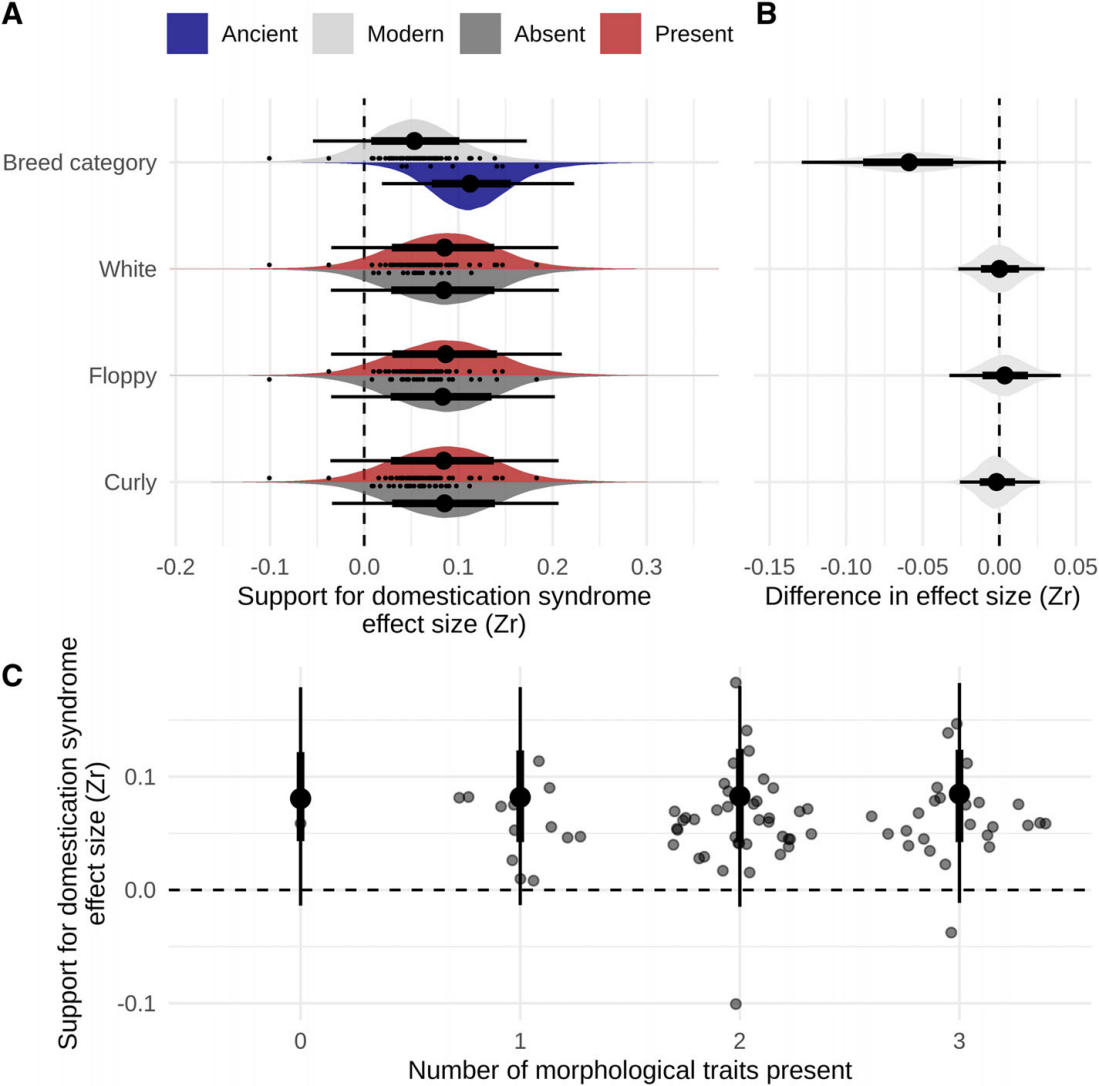
Morphological traits and the strength of behavioral correlations. (A) Estimated support for the DS, quantified as the strength of behavioral correlations (*Z*_r_) depending on the presence or absence of morphological traits (relaxed assessment) and trait category. (B) Regression coefficients indicating the difference between binary categories, as in (A). (C) The number a morphological traits present (relaxed assessment), that is, morphological score, related to the estimated strength of behavioral correlations within the DS. In all panels, density distributions depict the full posterior distributions, with the thick lines covering the 66% credible interval, thin lines the 95% credible interval, and point estimate the posterior median. Scattered points in (A) and (C) are the estimated average effect size per breed (as in Fig. 2).

Second, to test whether the presence of white pigmentation, floppy ears, and curly tails predicts the strength of any of the behavioral correlations, we evaluated these traits as binary predictors of DS support. We found that there was no difference in the behavioral correlations when any of the three morphological traits were present or absent (Tables 1, S3, and S4; Figs. 3A, 3B, and S2). We emphasize that there is no support for even a very small difference in effect size (most extreme effect within CI: 0.04; Table 1). We did not confirm an effect of breed age, as the difference between ancient and modern breeds could not be clearly distinguished from 0, although considerable uncertainty in this estimate remains and most of the posterior favors stronger behavioral correlations in ancient breeds (Figs. 3A and 3B; Table 1; for conservative measurements see Table S2).

**Table 1:** Predictive value of morphological traits. Predictive value of the presence or absence of morphological traits on the strength of behavioral correlations in the DS. Posterior mean, posterior standard deviation (SD), and 95% credible Interval (CI) given for breed category (ancient and modern) and the three morphological traits white pigmentation, floppy ears, and curly tail

Term	Posterior mean	Posterior SD	95CI _lower_	95CI _upper_
Intercept	0.113	0.053	0.020	0.220
Breed category	−0.060	0.034	−0.129	0.004
White pigmentation	0.001	0.014	−0.027	0.030
Floppy ears	0.004	0.019	−0.033	0.040
Curly tail	−0.001	0.014	−0.026	0.026

Last, we evaluated support for the DS based on the “morphology score” of each breed, which ranged from 0 to 3 depending on how many, if any, of the three morphological traits are present in a breed (Fig. 3C; Supplemental Files). We found that the number of morphological traits present in a breed did not predict the strength of behavioral correlations (0 traits: posterior mean_slope_ = 0.080, 95CI [–0.006, –0.191]; one trait: posterior mean_slope_ = 0.080, 95CI [–0.008, –0.193]; two traits: posterior mean_slope_ = 0.082, 95CI [–0.006, –0.192]; three traits: posterior mean_slope_ = 0.083, 95CI [–0.004, –0.190]). Given the small number of ancient breeds, we were not able to include breed age in this morphology score analysis.

## Discussion

Here, we tested whether the presence of three traits referred to as the morphological markers of domestication (white pigmentation, floppy ears, and curly tails) predicted the strength of behavioral correlations within the DS. Contrary to the expectations of covariation among DS traits, we found that these morphological traits did not covary among themselves, nor did they predict the strength of behavioral correlations among dog breeds. Further, the number of morphological traits in a breed did not predict the strength of behavioral correlations. Additionally, we found no effect of breed age, that is, ancient and modern breeds, in the predictive value of morphological traits on behavioral correlations. A high covariance among DS traits suggests a strong, central role for their shared origin in a single developmental source (e.g., white pigmentation arising as a by‐product of increased tameness, Wilkins et al. 2014), whereas a lack of covariance suggests a more complex genotype to phenotype relationship. Thus, the lack of covariation among morphological and behavioral traits in our study is not consistent with the hypothesis that trait alterations in the DS are founded in a singular developmental source (Belyaev 1979; Wilkins et al. 2014).

The DS in animals is primarily based on observations in present day domesticates. However, the ability of phenotypes in extant domesticates to provide insights about altered selection pressures during initial domestication is complicated by postdomestication selection events, that is, improvement traits (Olsen and Wendel 2013; Larson and Fuller 2014, Lord et al. 2019). Initial stages of domestication likely acted upon existing variation at multiple loci across the genome (Larson and Fuller 2014), but the breed‐specific morphology and behavior expressed in present day dog breeds were likely selected for postdomestication during breed formation. Many of the morphological traits seen across modern dog breeds can therefore not be assumed to be domestication traits. Rather they are most likely improvement traits. Thus, although studies refer to the phenotypes of modern dog breeds as evidence for the DS (Wilkins et al. 2014; Sánchez‐Villagra et al. 2016), whether these traits are relevant to domestication itself is questionable. Our findings of a lack of covariation among morphological and behavioral traits, rather than providing insights into the DS, therefore could be due to these traits being improvement traits, for which no covariance is expected. Regardless, the phenotypes of modern dog breeds should be interpreted with caution when trying to understand the domestication process.

One way to gain more insight into selection pressures during earlier stages of dog domestication, rather than those of breed improvement, is to include a temporal comparison by separating out ancient breeds and modern breeds. Here, we investigated whether the presence of morphological traits predict the strength of behavioral correlations in each breed group, but could not confirm such an effect. Given that selection on tameness alone can generate the DS in foxes (Trut et al. 2009), and that aggression shows selective signatures directly associated with altered selection pressures during initial domestication stages in these selection lines of foxes (Kukekova et al. 2018), it is likely that initial selection pressures during dog domestication acted upon behavior, not morphology (sensu Belyaev et al. 1985; Trut et al. 2009). Thus, with behaviors in the DS likely representing domestication traits, behavioral domestication phenotypes might to a larger extent be maintained in ancient compared to modern breeds. Morphology in dog breeds on the other hand is arguably linked to breed improvement (Larson and Fuller 2014), as reflected in the large variability in morphological trait combinations across dog breeds as quantified here.

In sum, whether the lack of covariance between morphology and behavior in dogs is due to decoupling of independent domestication alleles (possibly caused by altered selection regimes during breed formation), these traits never having covaried, or whether it is because we are applying a domestication hypothesis on traits that are not actual domestication traits, but rather improvement traits, remains an open question. If the latter is true, which seems likely for dogs, we must reevaluate our expectations of the DS and thereby also our assessment of DS traits in present day domesticates, as they have limited bearing of the initial selection pressures applied during domestication (Lord et al. 2019). Including contemporary populations of primitive canids, such as dingoes (*Canis dingo*), in future research efforts could provide further insight into the consequences of domestication, as they likely reflect the altered selection pressures during early stages domestication (Smith et al. 2017) without significant signatures of later artificial selection for improvement traits.

## CONFLICT OF INTEREST

The authors declare no conflict of interest.

Associate Editor: J. Slate

## PHOTO REFERENCES

English Springer Spaniel: By Kruming ‐ Own work, CC BY‐SA 3.0, https://commons.wikimedia.org/w/index.php?curid=18855288

Staffordshire Bull Terrier: By Spcenter ‐ Own work, CC BY‐SA 3.0, https://commons.wikimedia.org/w/index.php?curid=7662830

Shiba Inu: By Roberto Vasarri ‐ Own work, Public Domain, https://commons.wikimedia.org/w/index.php?curid=5788123

Labrador retriever: Own work Date: 20.1.2006 Author: Herwig Kavallar, https://commons.wikimedia.org/w/index.php?curid=744735

Alaskan malamute: By SCMW ‐ Own work, CC BY 3.0, https://commons.wikimedia.org/w/index.php?curid=3329851

Siberian husky: By Utopialand ‐ self‐made (http://www.utopialands.com), CC BY‐SA 4.0, https://commons.wikimedia.org/w/index.php?curid=3270359

Pug: By Cwazi at Dutch Wikipedia (Original text: Bonnie van den Born, http://www.bonfoto.nl)—transferred from nl.wikipedia to Commons.(Original text: Eigen werk/own work), CC BY‐SA 3.0, https://commons.wikimedia.org/w/index.php?curid=1861840

Dalmatian: By Dalmatiner24.eu ‐ Dalmatiner24.eu, Public Domain, https://commons.wikimedia.org/w/index.php?curid=12558543

German shorthaired pointer: By Bonnie van den Born, http://www.bonfoto.nl—transferred from nl.wikipedia to Commons., CC BY‐SA 3.0, https://commons.wikimedia.org/w/index.php?curid=1745399

Bernese Mountain Dog: By AnetaAp ‐ https://commons.wikimedia.org/wiki/File:Bernese_Mountain_DOg.jpg, CC BY‐SA 4.0, https://commons.wikimedia.org/w/index.php?curid=63333376

Rhodesian Ridgeback: By Bonnie van den Born, http://www.bonfoto.nl—transferred from nl.wikipedia to Commons., CC BY‐SA 3.0, https://commons.wikimedia.org/w/index.php?curid=1747204

St. Bernard: By Cassie J ‐ originally posted to Flickr as Harley‐parents' dog, CC BY 2.0, https://commons.wikimedia.org/w/index.php?curid=5120324

## Supporting information


**Figure S1**. Morphological scores on dog phylogeny (conservative).
**Figure S2**. Predictive value of morphological traits on individual behavioral correlations (relaxed).

Supplementary Material

**Table S1**. Predictive value of morphological traits, when Saluki is coded as a modern breed.
**Table S2**. Predictive value of morphological traits (conservative).
**Table S3**. Predictive value of morphological traits on individual behavioral correlations, output (relaxed).
**Table S4**. Predictive value of morphological traits on individual behavioral correlations, output (conservative).
