# Supplementary material for: Morphology does not covary with predicted behavioral correlations of the domestication syndrome in dogs
Source: Evol Lett. 2020 Apr 10;4(3):189–99. doi: 10.1002/evl3.168 (PMC7293089; doi:10.1002/evl3.168)
Supplement: Supplementary file 2 — Supplementary Material [file EVL3-4-189-s002.pdf]

Impact summary

Domesticated animals display suites of altered morphological, behavioural and physiological traits compared to their wild ancestors, a phenomenon known as the domestication syndrome (DS). Classic morphological “domestication traits” are white pigmentation, floppy ears and curly tails and reduced aggression and increased sociability are among the expected behavioural changes caused by domestication. Because these alterations are observed to co-occur across a wide range of present day domesticates, the traits within the DS are assumed to covary within species and a single developmental mechanism has been suggested to cause the DS. However, very few studies have tested whether the traits within DS actually covary. The domestic dog has been argued to be the only species expressing the full DS, but dogs have been bred for highly breed-specific morphological and behavioural traits and key behavioural and morphological DS traits do not appear to occur simultaneously across breeds. It is therefore unclear if we should expect the DS in dogs. Here we investigated the relationship between classic morphological DS traits and behavioural correlations in the DS in 78 dog breeds. Contrary to the expectations, we found that morphological traits did not covary among themselves, nor did they predict the strength of behavioural correlations among dog breeds. Further, the number of morphological traits in a breed did not predict the strength of behavioural correlations. Our results thus contrast with the hypothesis that the DS arises due to a shared underlying mechanism, but more importantly, questions if the morphological traits embedded in the DS are actual domestication traits or post-domestication improvement traits. For dogs, it seems highly likely that strong selection for breed specific morphological traits only happened recently and in relation to breed formation. Present day dogs therefore have limited bearing of the initial selection pressures applied during domestication and we should re-evaluate our expectations of the DS accordingly.
